# Toward Understanding the Sex Differences in the Biological Mechanism of Social Stress in Mouse Models

**DOI:** 10.3389/fpsyt.2021.644161

**Published:** 2021-02-16

**Authors:** Aki Takahashi

**Affiliations:** Laboratory of Behavioral Neuroendocrinology, Faculty of Human Sciences, University of Tsukuba, Tsukuba, Japan

**Keywords:** social stress, female, mouse, sex difference, repeated social defeat stress model (RSDS)

## Abstract

Significant sex differences in terms of prevalence, symptomatic profiles, severity, and comorbidities of psychiatric disorders are quite common. Women have been shown to be more vulnerable to stress and are nearly twice as likely as men to develop stress-related disorders such as depression and anxiety. Therefore, understanding sex differences with respect to the neurobiological mechanisms underlying stress-related disorders is important for developing more efficient pharmacological interventions for women. However, most preclinical studies on stress-related disorders have focused heavily on male rodents. Here, recent developments in the study of repeated social defeat stress models in female mice are summarized. Our findings suggest that a variety of factors need to be considered when employing this model.

Chronic subordination and repeated defeat experiences induce prolonged stress responses and cause several stress-related behaviors, such as exaggerated anxiety, social avoidance, anhedonia, and behavioral despair (1–4). Thus, the repeated social defeat stress (RSDS) model has been widely employed for studying neurobiological mechanisms underlying stress-related disorders. RSDS has long-term effects on stress-related behaviors, which are reversed by chronic—but not acute—treatment with antidepressants (5). Importantly, the RSDS model allows researchers to study the individual differences in stress susceptibility, and about half of the socially defeated animals show depressive-like symptoms (6). Therefore, although several models of depression exist (i.e., forced swim test, tail suspension test, chronic mild stress, uncontrollable stress, and olfactory bulbectomy), the RSDS model is considered to have high etiological, predictive, and face validity (7). However, RSDS studies have traditionally been restricted to males because this model relies on intermale territorial aggression. Female mice and rats are considered to have low levels of aggression, except when they are mothers (maternal aggression), and male-to-female aggression is rare in these species. Recently, efforts have been made to establish female RSDS models in mice because they are considered malleable to genetic modification and other neurobiological techniques (Table 1).

**Table 1:** Female social defeat stress mouse models.

	**Male to female**					**Female to female**		**Witness**
Manipulation	Gq-DREADD activation of the VMHvl of aggressor male by CNO i.p. injection (2)			Application of male urine to the base of the tail and the vaginal orifice of test female (6)	Simultaneous intoduction of male and female C57BL/6J into aggressor's homecage (7)	Pair housed female aggressor with a male (either intact or castrated) (8)		Witnessing social defeat in other male C57BL/6 (9)
Subject	C57BL/6J female			C57BL/6J female	C57BL/6J female	C57BL/6J female		C57BL/6 female
Age	8 weeks			3–4 months	8 weeks	12 weeks		10 weeks
Aggressor	ERα-Cre male mice with hM3D expression in the VMHvl			CD1 male	CD1 male	CFW female		CD1 male attack C57BL/6 male
Defeat duration	10 min, 10 day	5 min, 10 day		5 min, 10 day	5 min, 10 days	Acute: 5 min, 1day	Chronic: 5 min, 10day	10 min, 10 days
Attacked days	ave 9.6 days			median 5 days	64.33% of interaction			0
**Housing condition**	**Sensory contact**	**Individual housing**	**Paired with another female**	**Sensory contact**	**Sensory contact**	**Individual housing**	**Sensory contact**	**Sensory contact**
Social interaction test	↓	↓	↓	↓	↓	-	↓	↓
	S < R=C	S < R=C	S < R=C	S < R=C	S < R=C	(direct interaction)		
Rate of susceptible	10–19%	50%	50%	58%	About 63%	na	na	na
Effect of estrous cycle	-	-	-	-	-	na	na	na
Anxiety-like behavior	na	-^*^	↑	↑	↑	na	-	(↑)
			S=R>C	S=R>C	S>R=C			
Anhedonia	na	na	na	↑	↑	na	na	↑
				S>R=C	S>R=C			
Body weight	na	-	↓	na	na	na	-	↓
			S < R=C					
Corticosterone	na	na	na	↑	↑	↑	↑	↑
					S>R=C			

* *No effect of RSDS due to a reduction of body weight and an increased anxiety-like behavior in control animals by individual housing*.

*Anhedonia: reduction of sucrose preference, Sensory contact: Housed with an aggressor male via perforated divider. Susceptible and resilient were defined by social interaction behavior in the social interaction test. S, susceptible; R, resilient; C, control. Effect of estrus cycle indicates its effect on social interaction behavior*.

*↑, increase; ↓, decrease; -, no effect; na, not tested*.

One approach involved artificially inducing female-directed aggression by manipulating the activity of the hypothalamic attack area of aggressor animals. Following the chemogenetic activation of estrogen receptor alpha (ERα)-positive neurons in the ventrolateral subdivision of the ventromedial hypothalamus (VMHvl), the male aggressor mice exhibited constant and intense aggressive behavior toward females, which was comparable to inter-male aggression (8). Defeated females in this model exhibited social avoidance and increased anxiety-like behaviors (8, 9). Importantly, consistent with the findings in males (6), large individual differences in social avoidance behavior were observed, and only the females that showed social avoidance—and were thus considered stress-susceptible—exhibited a reduction in body weight and increased expression of proinflammatory cytokines (8). Therefore, this model enables the investigation of the mechanisms underlying stress susceptibility and resilience in both males and females. Similarly, surgical lesions in the mediobasal hypothalamus of female rats induced aggressive behavior toward female intruders (10). However, this inter-female RSDS had a relatively mild effect on stress responses in defeated female rats, and they were more vulnerable to social instability stress, wherein social isolation and crowding stress were combined (10).

Another method uses male pheromones to induce male-to-female aggression. In one model, male urine was applied on the body of target female mice (11). Although the level of aggressive behavior toward urine-applied females was lower than that observed in intermale aggression, the application of urine induced similar behavioral changes in both sexes, including social avoidance and increased anxiety-like behaviors. Another method involved placing both male and female intruders simultaneously inside the territory of aggressor male mice, inducing non-specific aggressive behavior toward intruders of both sexes (12). Again, while female intruders in this model experienced fewer attacks than males, both sexes showed similar significant social avoidance, increased anxiety-like behaviors, and elevated blood corticosterone levels, particularly in susceptible animals.

More researchers are trying to develop more ethologically-natural models without any artificial intervention. For example, a recent study showed that a subpopulation of female mice (about 65%) exhibited aggressive behaviors toward female as rival aggression, which was comparable to those of males (13). When a female mouse was housed with a male mouse, even a castrated one, the resident females showed rival aggression toward the intruding female. RSDS with this interfemale aggression increased corticosterone levels, reduced social interaction and social hyperthermia, and disrupted nest building. In contrast, these females did not undergo changes in body weight or anxiety-like behaviors (13). Furthermore, witnessing defeat in other animals has been shown to induce depression-like behaviors, increase corticosterone, and reduce body weight in both male and female mice and rats (14, 15).

Using these models, RSDS has been shown to activate the immune system in a similar manner in male and female mice, including splenomegaly, increased myelopoiesis, and accumulation of monocytes in the spleen and brain (9). In addition, stress-susceptible females showed higher interleukin 6 (IL-6) levels compared with resilient and control female mice, consistent with findings in male mice (8). However, other cytokine responses differed between sexes. For example, female—but not male—mice exhibited significantly elevated levels of proinflammatory cytokines, such as IL-1α, IL-1β, IL-12, and TNF-α (in addition to IL-6), following the first defeat, indicating a broader inflammatory profile in females following a single defeat episode (16). In addition, gene expression profiles in the prefrontal cortex following RSDS differed between the sexes, with males showing approximately twice as many differentially expressed genes in response to RSDS compared with females (16). Thus, although the main biological pathway for social stress seems to be shared between males and females, there are some important distinctions between the sexes. One study using an RSDS mouse model showed that a long non-coding RNA whose expression is downregulated specifically in women with depression has antidepressant effects in female—but not male—mice (17). Therefore, the female RSDS model will provide important insights into sex differences in the mechanism of stress susceptibility, which will lead to the development of more efficient pharmacological interventions for stress-related disorders in women.

However, there are some factors that need to be considered when using the female RSDS model. It is likely that housing conditions have different effects on males and females. In males, sensory exposure to a dominant male aggressor over a perforated divider after physical defeat stress has been shown to enhance susceptibility; however, in females, this sensory exposure to an aggressor male induced a stress-resilient phenotype (8). However, social isolation imposed via individual housing strongly induced stress in female mice, but not in male mice, and even control females (without RSDS) in individual housing showed higher anxiety-like behaviors compared with group-housed females (8). Thus, there are important differences with respect to the effects of various social stresses or *stressors* (e.g., social defeat, social instability, sensory contact, and social isolation) on the sexes. In addition, the effects may also vary depending on whether the female is attacked by males or females. Inter-female aggression seems to have a milder effect on female behavior and physiology than male-to-female aggression. These facts make it difficult to directly compare stress susceptibility between males and females. To study the molecular and cellular mechanisms underlying social stress in female mice, appropriate test conditions that induce high levels of stress in females must be explored. Another important factor to be considered in the female model is the estrous cycle. However, in female RSDS models, the estrous cycle did not affect social avoidance or anxiety-like behaviors (Table 1). In addition, while prolonged stress causes the disruption of the estrous cycle in humans, disruption of the estrous cycle was not observed in the inter-female RSDS model in mice (13), and this aspect must therefore be examined in other models.

Although this is just the beginning of the employment of a female RSDS model to study the mechanisms underlying social stress and stress susceptibility in female mice, additional studies utilizing these models are expected to uncover important targets for the treatment of stress-related disorders in women.

## Data Availability Statement

The original contributions presented in the study are included in the article/supplementary material, further inquiries can be directed to the corresponding authors.

## Author Contributions

The author confirms being the sole contributor of this work and has approved it for publication.

## Conflict of Interest

The author declares that the research was conducted in the absence of any commercial or financial relationships that could be construed as a potential conflict of interest.
